# Medicine

**Published:** 1899-03

**Authors:** Albert T. Lytle

**Affiliations:** Buffalo, N. Y.


### Progress in Medical Science.

### MEDICINE.

CONDUCTED BY ALBERT T. LYTLE, M. D., Buffalo, N. Y.

### DIPHTHERIA ANTITOXIN.

D. C. MORIARTA (*N. Y. Med. Jour.*), in a paper on diphtheria antitoxin, read before the Medical Society of Saratoga Springs, N. Y., concludes as follows: After analysing a series of 207 cases, (*a*) Diphtheria antitoxin (*per se*) is harmless; (*b*) Diphtheria antitoxin is practically a specific in diphtheria; (*c*) Diphtheria antitoxin is the rational treatment for diphtheria; (*d*) Diphtheria antitoxin must be used early; (*e*) Diphtheria antitoxin must be used in full dose; (*f*) It is necessary to have a reliable product; (*g*) Intubation is an essential associate of antitoxin in laryngeal cases; (*h*) There is no case so far advanced that antitoxin should not be used; (*i*) We should not wait for the report of the bacteriologist, but use it promptly on clinical grounds; (*j*) It must not be the last resort, nor can it be of much service in small doses.

### TUBERCULAR BACILLI ON GRASSES.

MÖLLER (*Deutsche Med. Woch.*, No. 24,) reports discovering on grasses and in the feces of the domestic animals a bacillus resembling that of tuberculosis in size, shape and in action toward acids and alcohol. Care was made to eliminate tubercular trouble in the animals. They differ in their growth upon various media. Inoculation of guinea-pigs showed infiltration and caseation at point of inoculation; lymph glands enlarged; in mesentery and peritoneum numerous nodules, whitish in color; in lungs, similar nodules; in all, the changes resembled those of experimental tuberculosis.

### CHOLELITHIASIS IN INFANCY.

A. V. WENDEL, (*Medical Record*, June, 1898,) in a paper on Cholelithiasis in infancy and in childhood, read before the New Jersey Surgical Society, speaking of xiphoid pain as a diagnostic symptom, says: "Rubini was the first to call especial attention to pain around the xiphoid cartilage from gall-stones during their expulsion. We have frequently noticed this symptom during the transition of calculi through the ducts, particularly in calculous incarceration of the cystic duct. We have observed this symptom in six out of eight cases of calculous obstruction of the cystic duct in adults, and the localisation was proved by operation. Rubini's symptom certainly deserves more attention, because if extended observation should prove the reliability of it as a sign of obstruction of the cystic duct, then one of the perplexing questions in the surgical diagnosis of cholelithiasis will be rendered more easy of solution. Permanent cystic obstruction is well known often to result in serious or even fatal complications. These cases can be properly treated only by surgical intervention, and, as our diagnostic prehension of obstruction of the cystic duct is not so satisfactory as the development of the oprative technique for this condition, more ought to be heard of Rubini's xiphoidal pain."

### BORAX IN EPILEPSY.

C. E. TODD, (*Australia Med. Gaz.*, July, 1898,) from the result of administering ten grains of borax four times a day in each of five cases of epilepsy, believes he caused the convulsions to cease. One patient remained well for one year.

### LANDRY'S PARALYSIS.

C. M. GREENE (*Philadelphia Med. Jour.*, No. 49,) reports in detail the clinical course of a case of this kind, in which artificial respiration was maintained for forty-one days, the post mortem and the histologic findings in the case. The pathologic process was a parenchymatous ascending anterior poliomyelitis, involving the whole length of the cord and extending into the medulla and pons; there was degeneration present in the peripheral nerves and in the kidneys. Greene believes that in this case the development must be ascribed to auto-intoxication of intestinal origin; the girl's descending colon being loaded to the spleenic flexure at least with impacted feces, consisting largely of masses of grape-skins.

